# Crystal structure and Hirshfeld surface analysis of 4-[4-(1*H*-benzo[*d*]imidazol-2-yl)phen­oxy]phthalo­nitrile monohydrate

**DOI:** 10.1107/S2056989018008745

**Published:** 2018-06-19

**Authors:** Pinar Sen, Sevgi Kansiz, Necmi Dege, Turganbay S. Iskenderov, S. Zeki Yildiz

**Affiliations:** a Uskudar University, Faculty of Engineering and Natural Sciences, Department of Forensic Science, 34662, Istanbul, Turkey; b Ondokuz Mayıs University, Faculty of Arts and Sciences, Department of Physics, 55139, Kurupelit, Samsun, Turkey; c Taras Shevchenko National University of Kyiv, Department of Chemistry, 64 Vladimirska Str., Kiev 01601, Ukraine; d Sakarya University, Faculty of Arts and Sciences, Department of Chemistry, 54187 Sakarya, Turkey

**Keywords:** crystal structure, benzimidazole, hydrogen bonding, Hirshfeld surfaces

## Abstract

In the title compound, C_21_H_12_N_4_O·H_2_O, the five-membered ring is essentially planar with a maximum deviation of 0.004 (2) Å. An N—H⋯O hydrogen bond connects the organic and water mol­ecules. In the crystal, O—H⋯N hydrogen bonds link mol­ecules into a two-dimensional network parallel to (100). Hirshfeld surface analyses and two-dimensional fingerprint plots were used to qu­antify the inter­molecular inter­actions present in the crystal, indicating that the most important contributions for the crystal packing are from H⋯H (28.7%), C⋯H/H⋯C (27.1%), N⋯H/H⋯N (26.4%), C⋯N/N⋯C (6.1%), O⋯H/H⋯O (3.7%) and C⋯C (6.0%) inter­actions.

## Abstract 2

In the crystal, strong O—H⋯N hydrogen bonds link the mol­ecules into supra­mol­ecular chains propagating along the *c-*axis direction.

## Chemical context   

Benzimidazole derivatives, as nitro­gen-containing aromatic heterocyclic compounds, are a very important class owing to their biological importance (Preston, 2008 ▸). They are widely used as anti­ulcer, anti­fungal and anti­mycobacterial compounds (Patil *et al.*, 2008 ▸) and have also attracted attention as organic fluorescent chromophores in recent years (Verdasco *et al.*, 1995 ▸). Phthalo­nitrile derivatives are widely used precursors for the preparation of phthalocyanines, an important class of mol­ecules not only as commercial pigments but also as important functional materials in many areas (Sharman *et al.*, 2003 ▸). The preparation of phthalocyanines is carried out by cyclo­tetra­merization reactions of phthalo­nitriles. The development of benzimidazole derivative-substituted phthalocyanines from the related phthalo­nitriles is crucial in terms of achieving a combination of functional groups.
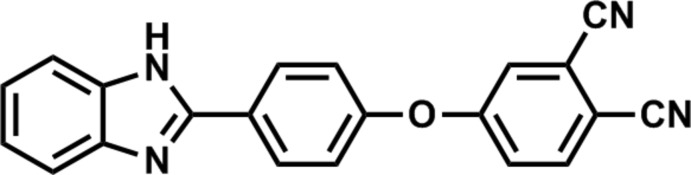


We now report for the first time that benzimidazole groups linked directly through oxygen bridges to phthalo­nitrile units are new functionalized materials. We have described the synthesis, characterization and spectroscopic behavior of the synthesized starting phthalo­nitrile compound (Sen *et al.*, 2018 ▸).

## Structural commentary   

**Figure 1:**
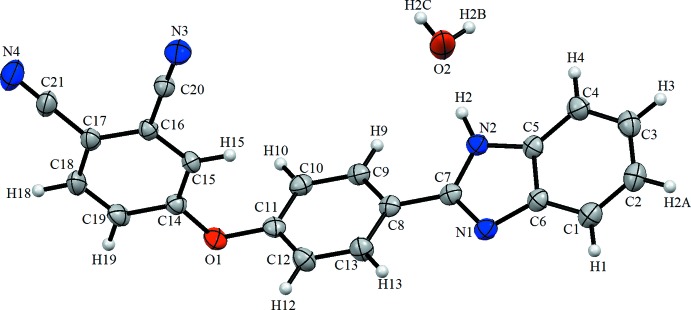
The mol­ecular structure of the title compound, showing the atom labelling. Displacement ellipsoids are drawn at the 20% probability level.

The asymmetric unit of the title compound contains one independent mol­ecule and one water mol­ecule (Fig. 1 ▸). The five-membered ring is essentially planar with maximum deviations of 0.004 (2) Å for atom N2 and −0.004 (2) Å for C5 and the N1=C7, N1—C6 and C5—C6 bond lengths are 1.324 (3), 1.388 (3) and 1.391 (3) Å, respectively. The dihedral angle between the fused C1–C6 and C5/N2/C7/N1/C6 rings is 1.71 (13)° while the C8–C13 ring subtends a dihedral angle of 16.03 (12)° with the C5/N2/C7/N1/C6 ring plane. The C14–C19 ring makes a dihedral angle of 83.55 (11)° with the C8–C13 ring.

## Supra­molecular features   

**Figure 2:**
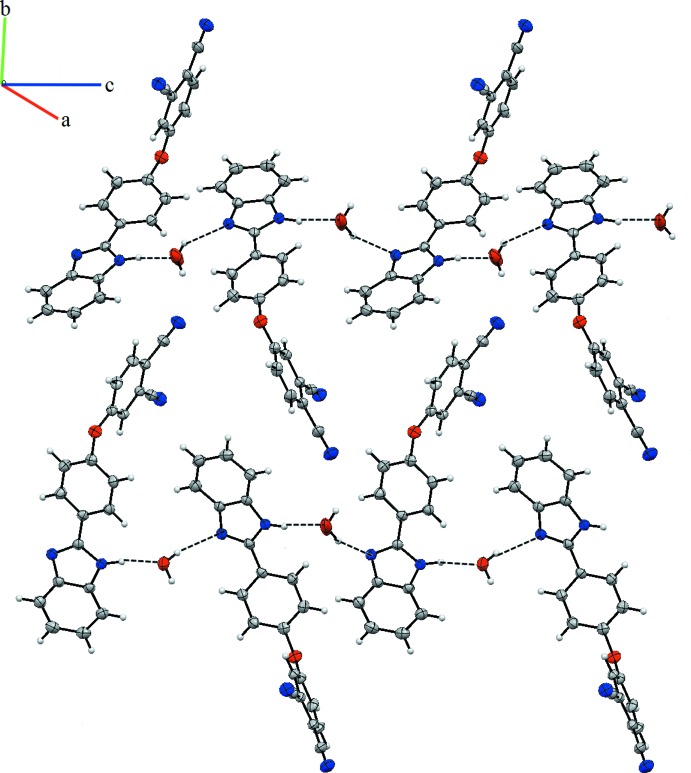
A partial view of the crystal packing. Dashed lines denote the inter­molecular N—H⋯O and O—H⋯N hydrogen bonding.

In the crystal, an N2—H2⋯O2 hydrogen bond connects the organic and water mol­ecules (Table 1 ▸). O2—H2*C*⋯N1 hydrogen bonds connect the mol­ecules into a two-dimensional network parallel to (100) (Table 1 ▸, Fig. 2 ▸).

## Hirshfeld surface analysis   

**Figure 3:**
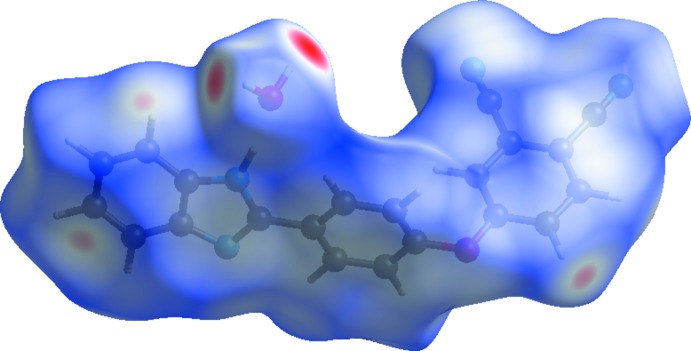
The Hirshfeld surface mapped over *d*_norm_.

*Crystal Explorer17.5* (Turner *et al.*, 2017 ▸) was used to analyse the inter­actions in the crystal. Figs. 3 ▸ and 4 ▸ show the Hirshfeld surfaces mapped over *d*_norm_. with a fixed colour scale of −0.4353 (red) to 1.4359 (blue) a.u. where red spots indicate the regions of donor–acceptor inter­actions (Aydemir *et al.*, 2018 ▸; Kansiz *et al.*, 2018 ▸; Şen *et al.*, 2017 ▸; Gümüş *et al.*, 2018 ▸)·There are five red spots in the *d*_norm_ surface (Fig. 3 ▸); these represent the N-acceptor atoms involved in the inter­actions listed in Table 1 ▸.

**Figure 5:**
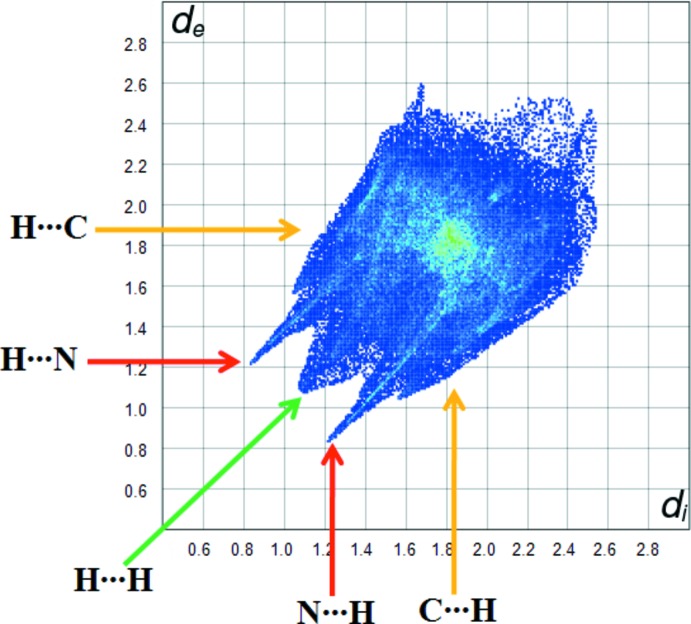
Fingerprint plot for the title compound.

**Figure 6:**
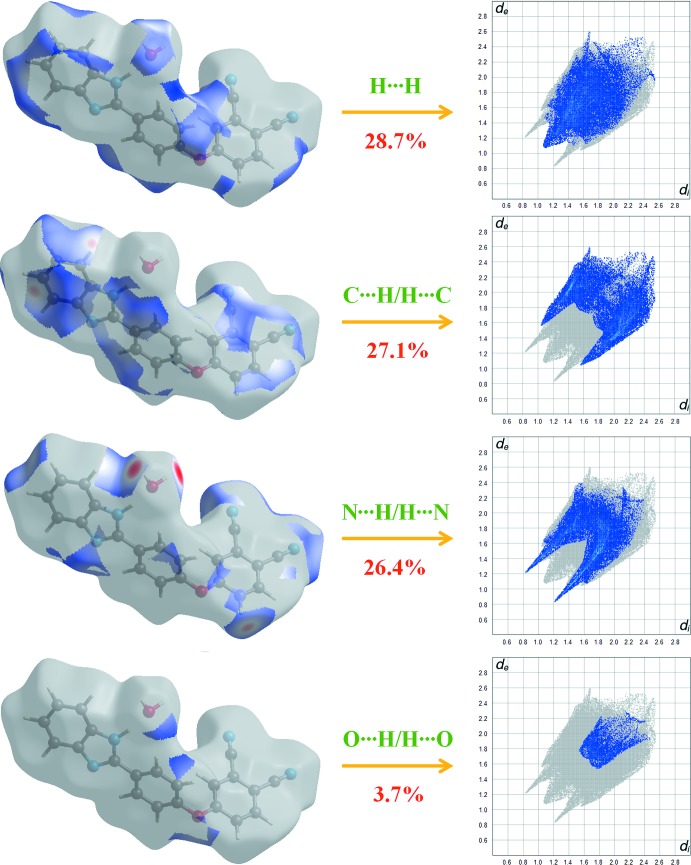
Two-dimensional fingerprint plots with a *d*_norm_ view for the H⋯H (28.7%), C⋯H/H⋯C (27.1%), N⋯H/H⋯N (26.4%) and O⋯H/H⋯O (3.7%) contacts in the title compound.

The overall two-dimensional fingerprint plot and those showing different contacts are characterized in Fig. 5 ▸, together with their relative contributions to the Hirshfeld surface. H⋯H/H⋯H inter­actions, contributing 28.7% to the overall crystal packing, are some of the important inter­actions, and are shown in Fig. 6 ▸ as an end point that points to the origin with the tips at *d*_i_ = *d*_e_ = 1.1 Å. The C⋯H/H⋯C contacts in the structure, with a 27.1% contribution to the Hirshfeld surface, have a symmetrical distribution of points, with the tips at *d*_e_ + *d*_i_ = 2.7 Å. The contribution from the N⋯H/H⋯N contacts, corresponding to C—H⋯N and O—H⋯N inter­actions, is represented by a pair of sharp spikes characteristic of a strong hydrogen-bond inter­action (26.4%). The O⋯H/H⋯O contacts, with a 3.7% contribution, appear with the points of low densities. Lastly, the C⋯N/N⋯C, C⋯C/C⋯C and O⋯C/C⋯O inter­actions in the structure with 6.1, 5.5 and 1.4% contributions, respectively, have symmetrical distributions of points.

## Database survey   

There are no direct precedents for the structure of the title compound in the crystallographic literature (Groom *et al.*, 2016 ▸). However, there are several precedents for the 2-(4-hy­droxy­phen­yl)benzimidazole, including 4-[4-(1-allyl-1*H*-benzo[*d*]imidazole-2­yl)phen­oxy]phthalo­nitrile (Sen *et al.*, 2018 ▸), 4-(1*H*-benzo[d]imidazol-2-yl)phenol (Zhan *et al.*, 2007 ▸), 2-(4-meth­oxy­phen­yl)-1*H*-benzimidazole (Moreno-Diaz *et al.*, 2006 ▸) and 4-(1*H*-benzimidazol-2-yl)phenol (Zhou *et al.*, 2006 ▸).

## Synthesis and crystallization   

**Figure 7:**
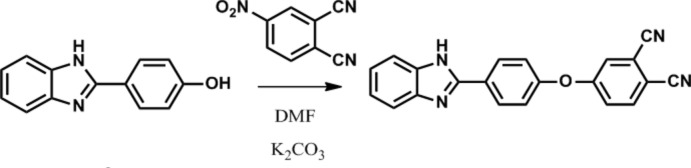
The synthesis of the title compound.

The synthesis of the title compound (Fig. 7 ▸) was described by Sen *et al.* (2018 ▸). 4-[4-(1*H*-Benzo[*d*]imidazole-2­yl)phen­oxy]phthalo­nitrile, 4-nitro­phthalo­nitrile (0.989 g, 5.71 mmol) and 2-(4-hy­droxy­phen­yl)benzimidazole (1.2 g, 5.71 mmol) were dissolved in DMF (15 mL) under argon. After stirring for 15 min, anhydrous K_2_CO_3_ (0.790 g, 5.71 mmol) was added portionwise over 2 h with efficient stirring. The suspension was maintained at 333 K for 24 h. The progress of the reaction was monitored by TLC using a CHCl_3_/EtOAc (10/1) solvent system. After the reaction was observed to be complete, the resulting mixture was poured into an ice–water mixture. The immediate precipitate was collected by filtration, washed with hot water, ethanol and diethyl ether and dried *in* vacuo. The desired pure compound was obtained in sufficient purity, yield: 96% (1.84 g). m.p. 421 K.

## Refinement   

Crystal data, data collection and structure refinement details are summarized in Table 2 ▸. The C-bound hydrogen atoms were included in calculated positions with C—H = 0.93 Å (aromatic) and allowed to ride, with *U*_iso_(H) = 1.2*U*_eq_(C).

## Supplementary Material

Crystal structure: contains datablock(s) I. DOI: 10.1107/S2056989018008745/qm2124sup1.cif

Structure factors: contains datablock(s) I. DOI: 10.1107/S2056989018008745/qm2124Isup2.hkl

Supporting information file. DOI: 10.1107/S2056989018008745/qm2124Isup3.cml

CCDC reference: 1849358

Additional supporting information:  crystallographic information; 3D view; checkCIF report

## Figures and Tables

**Figure 4:**
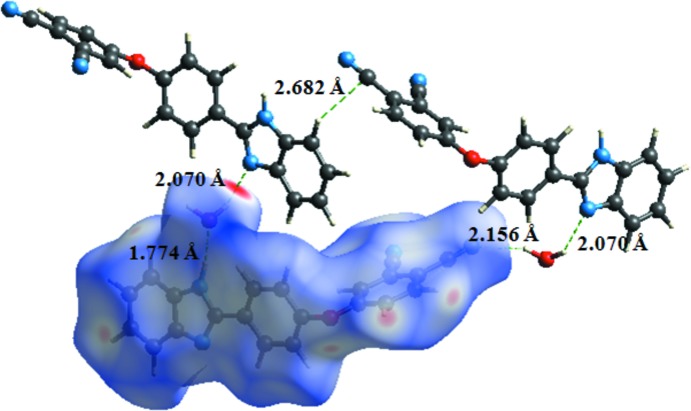
Hirshfeld surfaces mapped over *d*_norm_ to visualize the inter­molecular inter­actions.

**Table 1:** Hydrogen-bond geometry (Å, °)

*D*—H⋯*A*	*D*—H	H⋯*A*	*D*⋯*A*	*D*—H⋯*A*
N2—H2⋯O2	0.92 (2)	1.86 (3)	2.774 (3)	171 (2)
O2—H2*C*⋯N1^i^	0.85	2.17	2.876 (2)	140

Symmetry code: (i) 
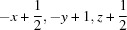
.

**Table 2:** Experimental details

Crystal data	
Chemical formula	C_21_H_12_N_4_O·H_2_O
*M*_r_	354.36
Crystal system, space group	Orthorhombic, *P* *b* *c* *a*
Temperature (K)	296
*a*, *b*, *c* (Å)	8.7657 (6), 27.285 (2), 14.6938 (13)
*V* (Å^3^)	3514.4 (5)
*Z*	8
Radiation type	Mo *K*α
μ (mm^−1^)	0.09
Crystal size (mm)	0.79 × 0.39 × 0.18

Data collection	
Diffractometer	STOE IPDS 2
Absorption correction	Integration (*X-RED32*; Stoe & Cie, 2004 ▸)
*T*_min_, *T*_max_	0.967, 0.988
No. of measured, independent and observed [*I* > 2σ(*I*)] reflections	33692, 3447, 1829
*R*_int_	0.079
(sin θ/λ)_max_ (Å^−1^)	0.617

Refinement	
*R*[*F* ^2^ > 2σ(*F* ^2^)], *wR*(*F* ^2^), *S*	0.044, 0.106, 0.91
No. of reflections	3447
No. of parameters	251
H-atom treatment	H atoms treated by a mixture of independent and constrained refinement
Δρ_max_, Δρ_min_ (e Å^−3^)	0.21, −0.25

Computer programs: *X-AREA* and *X-RED* (Stoe & Cie, 2004 ▸), *SHELXL2017/1* (Sheldrick, 2015 ▸), *ORTEP-3 for Windows* and *WinGX* (Farrugia, 2012 ▸) and *PLATON* (Spek, 2009 ▸).

## supplementary crystallographic information

### Crystal data

**Table:**

C_21_H_12_N_4_O·H_2_O	*D*_x_ = 1.339 Mg m^−^^3^
*M**_r_* = 354.36	Mo *K*α radiation, λ = 0.71073 Å
Orthorhombic, *P**b**c**a*	Cell parameters from 17763 reflections
*a* = 8.7657 (6) Å	θ = 1.4–27.6°
*b* = 27.285 (2) Å	µ = 0.09 mm^−^^1^
*c* = 14.6938 (13) Å	*T* = 296 K
*V* = 3514.4 (5) Å^3^	Stick, red
*Z* = 8	0.79 × 0.39 × 0.18 mm
*F*(000) = 1472	

### Data collection

**Table:**

STOE IPDS 2 diffractometer	3447 independent reflections
Radiation source: sealed X-ray tube, 12 x 0.4 mm long-fine focus	1829 reflections with *I* > 2σ(*I*)
Detector resolution: 6.67 pixels mm^-1^	*R*_int_ = 0.079
rotation method scans	θ_max_ = 26.0°, θ_min_ = 2.0°
Absorption correction: integration (X-RED32; Stoe & Cie, 2004)	*h* = −10→10
*T*_min_ = 0.967, *T*_max_ = 0.988	*k* = −33→33
33692 measured reflections	*l* = −18→18

### Refinement

**Table:**

Refinement on *F*^2^	0 restraints
Least-squares matrix: full	Hydrogen site location: mixed
*R*[*F*^2^ > 2σ(*F*^2^)] = 0.044	H atoms treated by a mixture of independent and constrained refinement
*wR*(*F*^2^) = 0.106	*w* = 1/[σ^2^(*F*_o_^2^) + (0.0484*P*)^2^] where *P* = (*F*_o_^2^ + 2*F*_c_^2^)/3
*S* = 0.91	(Δ/σ)_max_ < 0.001
3447 reflections	Δρ_max_ = 0.21 e Å^−^^3^
251 parameters	Δρ_min_ = −0.25 e Å^−^^3^

### Special details

**Table:**

Geometry. All esds (except the esd in the dihedral angle between two l.s. planes) are estimated using the full covariance matrix. The cell esds are taken into account individually in the estimation of esds in distances, angles and torsion angles; correlations between esds in cell parameters are only used when they are defined by crystal symmetry. An approximate (isotropic) treatment of cell esds is used for estimating esds involving l.s. planes.

### Fractional atomic coordinates and isotropic or equivalent isotropic displacement parameters (Å^2^)

**Table:**

	*x*	*y*	*z*	*U*_iso_*/*U*_eq_	
O1	0.69369 (16)	0.65950 (6)	0.29785 (11)	0.0737 (5)	
N2	0.2691 (2)	0.46625 (6)	0.26099 (13)	0.0612 (5)	
N1	0.3278 (2)	0.47874 (7)	0.11536 (11)	0.0660 (5)	
C15	0.4616 (2)	0.70114 (8)	0.34035 (13)	0.0556 (5)	
H15	0.401592	0.674922	0.321542	0.067*	
C8	0.4411 (2)	0.53611 (7)	0.22666 (14)	0.0570 (5)	
C16	0.3945 (2)	0.74273 (8)	0.37607 (13)	0.0555 (5)	
N3	0.1021 (3)	0.74647 (8)	0.38612 (15)	0.0877 (7)	
C11	0.6078 (2)	0.61778 (8)	0.27451 (15)	0.0617 (6)	
C7	0.3485 (2)	0.49377 (7)	0.20015 (14)	0.0575 (5)	
C14	0.6179 (3)	0.69884 (8)	0.33284 (14)	0.0595 (6)	
C5	0.1905 (3)	0.43125 (8)	0.21300 (14)	0.0598 (6)	
C6	0.2289 (3)	0.43919 (8)	0.12231 (15)	0.0626 (6)	
C20	0.2314 (3)	0.74515 (8)	0.38237 (15)	0.0629 (6)	
C9	0.4841 (3)	0.54391 (8)	0.31609 (14)	0.0636 (6)	
H9	0.455684	0.521444	0.360562	0.076*	
C17	0.4835 (3)	0.78215 (8)	0.40457 (14)	0.0641 (6)	
C10	0.5683 (3)	0.58453 (9)	0.34017 (15)	0.0669 (6)	
H10	0.597961	0.589294	0.400289	0.080*	
O2	0.2249 (4)	0.46945 (10)	0.44787 (13)	0.1486 (11)	
H2B	0.205614	0.441905	0.472290	0.223*	
H2C	0.175029	0.490023	0.479514	0.223*	
C13	0.4861 (3)	0.56973 (9)	0.16191 (15)	0.0728 (7)	
H13	0.460140	0.564674	0.101255	0.087*	
C19	0.7071 (3)	0.73832 (10)	0.35927 (16)	0.0730 (7)	
H19	0.812628	0.736903	0.353160	0.088*	
C18	0.6397 (3)	0.77925 (10)	0.39428 (16)	0.0796 (7)	
H18	0.700081	0.805688	0.411514	0.095*	
C1	0.1650 (3)	0.40948 (9)	0.05537 (17)	0.0779 (7)	
H1	0.189956	0.413927	−0.005580	0.093*	
C12	0.5683 (3)	0.61047 (9)	0.18562 (16)	0.0729 (7)	
H12	0.597142	0.633031	0.141412	0.087*	
C21	0.4137 (3)	0.82420 (10)	0.44552 (17)	0.0822 (8)	
C4	0.0891 (3)	0.39499 (8)	0.23910 (17)	0.0754 (7)	
H4	0.063662	0.390196	0.299896	0.090*	
N4	0.3579 (3)	0.85731 (10)	0.47866 (17)	0.1136 (9)	
C3	0.0277 (3)	0.36651 (9)	0.17176 (19)	0.0827 (7)	
H3	−0.040521	0.341761	0.187190	0.099*	
C2	0.0650 (3)	0.37372 (9)	0.08091 (19)	0.0822 (7)	
H2A	0.021084	0.353825	0.036701	0.099*	
H2	0.259 (3)	0.4705 (9)	0.3227 (17)	0.085 (8)*	

### Atomic displacement parameters (Å^2^)

**Table:**

	*U*^11^	*U*^22^	*U*^33^	*U*^12^	*U*^13^	*U*^23^
O1	0.0442 (9)	0.0814 (11)	0.0956 (11)	−0.0072 (9)	0.0038 (8)	−0.0091 (9)
N2	0.0693 (13)	0.0588 (11)	0.0555 (11)	0.0030 (9)	0.0003 (10)	0.0023 (10)
N1	0.0762 (13)	0.0634 (12)	0.0585 (11)	0.0056 (10)	0.0011 (10)	0.0022 (9)
C15	0.0494 (14)	0.0554 (13)	0.0620 (12)	−0.0078 (10)	−0.0041 (10)	−0.0002 (10)
C8	0.0545 (13)	0.0530 (13)	0.0633 (13)	0.0111 (11)	−0.0010 (10)	0.0039 (10)
C16	0.0581 (14)	0.0553 (13)	0.0532 (12)	−0.0051 (11)	−0.0036 (10)	0.0025 (10)
N3	0.0663 (15)	0.0929 (16)	0.1039 (17)	0.0086 (13)	0.0019 (13)	−0.0076 (12)
C11	0.0458 (13)	0.0665 (14)	0.0728 (15)	0.0047 (11)	0.0039 (11)	−0.0020 (12)
C7	0.0594 (14)	0.0550 (13)	0.0582 (13)	0.0133 (11)	−0.0004 (11)	0.0047 (10)
C14	0.0536 (15)	0.0676 (15)	0.0572 (12)	−0.0049 (12)	0.0003 (11)	−0.0017 (11)
C5	0.0635 (15)	0.0510 (12)	0.0651 (13)	0.0095 (12)	−0.0034 (11)	0.0033 (11)
C6	0.0676 (15)	0.0568 (13)	0.0632 (13)	0.0097 (12)	−0.0030 (12)	0.0003 (11)
C20	0.0639 (17)	0.0579 (14)	0.0668 (14)	0.0009 (12)	−0.0017 (13)	−0.0041 (11)
C9	0.0660 (15)	0.0651 (14)	0.0597 (13)	0.0066 (12)	0.0051 (11)	0.0027 (11)
C17	0.0725 (17)	0.0634 (15)	0.0563 (12)	−0.0144 (13)	−0.0031 (11)	−0.0020 (11)
C10	0.0606 (15)	0.0779 (16)	0.0620 (13)	0.0063 (13)	0.0017 (11)	−0.0049 (12)
O2	0.264 (3)	0.1188 (17)	0.0633 (11)	−0.079 (2)	0.0336 (15)	−0.0127 (11)
C13	0.0824 (17)	0.0731 (16)	0.0628 (13)	−0.0058 (14)	−0.0087 (13)	0.0085 (12)
C19	0.0528 (15)	0.098 (2)	0.0683 (14)	−0.0241 (14)	−0.0033 (11)	−0.0065 (13)
C18	0.085 (2)	0.0824 (18)	0.0709 (15)	−0.0335 (16)	−0.0013 (14)	−0.0168 (13)
C1	0.0890 (18)	0.0737 (16)	0.0710 (15)	0.0085 (15)	−0.0049 (14)	−0.0034 (13)
C12	0.0713 (17)	0.0743 (16)	0.0731 (15)	−0.0070 (13)	−0.0037 (13)	0.0125 (12)
C21	0.112 (2)	0.0648 (16)	0.0694 (15)	−0.0126 (16)	−0.0029 (14)	−0.0104 (13)
C4	0.0823 (18)	0.0674 (15)	0.0764 (15)	0.0009 (14)	0.0025 (14)	0.0061 (13)
N4	0.157 (2)	0.0810 (16)	0.1027 (18)	0.0009 (17)	−0.0028 (17)	−0.0252 (14)
C3	0.0792 (18)	0.0662 (16)	0.103 (2)	−0.0019 (13)	−0.0046 (16)	−0.0015 (15)
C2	0.087 (2)	0.0679 (17)	0.0922 (19)	0.0068 (15)	−0.0177 (16)	−0.0126 (14)

### Geometric parameters (Å, º)

**Table:**

O1—C14	1.363 (2)	C9—C10	1.378 (3)
O1—C11	1.407 (2)	C9—H9	0.9300
N2—C7	1.359 (3)	C17—C18	1.379 (3)
N2—C5	1.373 (3)	C17—C21	1.433 (4)
N2—H2	0.92 (2)	C10—H10	0.9300
N1—C7	1.324 (3)	O2—H2B	0.8500
N1—C6	1.388 (3)	O2—H2C	0.8500
C15—C14	1.376 (3)	C13—C12	1.370 (3)
C15—C16	1.382 (3)	C13—H13	0.9300
C15—H15	0.9300	C19—C18	1.364 (3)
C8—C13	1.379 (3)	C19—H19	0.9300
C8—C9	1.383 (3)	C18—H18	0.9300
C8—C7	1.465 (3)	C1—C2	1.364 (3)
C16—C17	1.393 (3)	C1—H1	0.9300
C16—C20	1.435 (3)	C12—H12	0.9300
N3—C20	1.135 (3)	C21—N4	1.137 (3)
C11—C12	1.366 (3)	C4—C3	1.368 (3)
C11—C10	1.369 (3)	C4—H4	0.9300
C14—C19	1.387 (3)	C3—C2	1.388 (4)
C5—C4	1.384 (3)	C3—H3	0.9300
C5—C6	1.391 (3)	C2—H2A	0.9300
C6—C1	1.392 (3)		
			
C14—O1—C11	117.92 (16)	C18—C17—C16	118.6 (2)
C7—N2—C5	107.66 (18)	C18—C17—C21	121.0 (2)
C7—N2—H2	129.0 (15)	C16—C17—C21	120.4 (2)
C5—N2—H2	123.1 (15)	C11—C10—C9	119.2 (2)
C7—N1—C6	104.91 (18)	C11—C10—H10	120.4
C14—C15—C16	119.5 (2)	C9—C10—H10	120.4
C14—C15—H15	120.3	H2B—O2—H2C	104.5
C16—C15—H15	120.3	C12—C13—C8	121.0 (2)
C13—C8—C9	118.4 (2)	C12—C13—H13	119.5
C13—C8—C7	119.96 (19)	C8—C13—H13	119.5
C9—C8—C7	121.66 (19)	C18—C19—C14	119.8 (2)
C15—C16—C17	120.7 (2)	C18—C19—H19	120.1
C15—C16—C20	119.1 (2)	C14—C19—H19	120.1
C17—C16—C20	120.2 (2)	C19—C18—C17	121.2 (2)
C12—C11—C10	120.9 (2)	C19—C18—H18	119.4
C12—C11—O1	119.1 (2)	C17—C18—H18	119.4
C10—C11—O1	120.0 (2)	C2—C1—C6	118.7 (2)
N1—C7—N2	112.18 (19)	C2—C1—H1	120.6
N1—C7—C8	124.8 (2)	C6—C1—H1	120.6
N2—C7—C8	122.99 (19)	C11—C12—C13	119.7 (2)
O1—C14—C15	123.5 (2)	C11—C12—H12	120.2
O1—C14—C19	116.3 (2)	C13—C12—H12	120.2
C15—C14—C19	120.2 (2)	N4—C21—C17	179.4 (3)
N2—C5—C4	132.6 (2)	C3—C4—C5	117.3 (2)
N2—C5—C6	105.2 (2)	C3—C4—H4	121.4
C4—C5—C6	122.1 (2)	C5—C4—H4	121.4
N1—C6—C5	110.03 (19)	C4—C3—C2	121.5 (2)
N1—C6—C1	130.7 (2)	C4—C3—H3	119.3
C5—C6—C1	119.3 (2)	C2—C3—H3	119.3
N3—C20—C16	178.8 (3)	C1—C2—C3	121.1 (2)
C10—C9—C8	120.9 (2)	C1—C2—H2A	119.4
C10—C9—H9	119.5	C3—C2—H2A	119.4
C8—C9—H9	119.5		
			
C14—C15—C16—C17	−0.1 (3)	C7—C8—C9—C10	−178.5 (2)
C14—C15—C16—C20	179.04 (19)	C15—C16—C17—C18	1.6 (3)
C14—O1—C11—C12	99.2 (2)	C20—C16—C17—C18	−177.6 (2)
C14—O1—C11—C10	−82.4 (2)	C15—C16—C17—C21	−177.0 (2)
C6—N1—C7—N2	0.1 (2)	C20—C16—C17—C21	3.8 (3)
C6—N1—C7—C8	−177.8 (2)	C12—C11—C10—C9	−1.7 (3)
C5—N2—C7—N1	−0.5 (2)	O1—C11—C10—C9	179.93 (19)
C5—N2—C7—C8	177.44 (19)	C8—C9—C10—C11	0.9 (3)
C13—C8—C7—N1	14.5 (3)	C9—C8—C13—C12	−1.4 (3)
C9—C8—C7—N1	−166.3 (2)	C7—C8—C13—C12	177.8 (2)
C13—C8—C7—N2	−163.2 (2)	O1—C14—C19—C18	179.5 (2)
C9—C8—C7—N2	16.0 (3)	C15—C14—C19—C18	1.1 (3)
C11—O1—C14—C15	−4.8 (3)	C14—C19—C18—C17	0.4 (4)
C11—O1—C14—C19	176.87 (19)	C16—C17—C18—C19	−1.7 (4)
C16—C15—C14—O1	−179.49 (18)	C21—C17—C18—C19	176.9 (2)
C16—C15—C14—C19	−1.2 (3)	N1—C6—C1—C2	−177.4 (2)
C7—N2—C5—C4	−177.5 (2)	C5—C6—C1—C2	0.7 (3)
C7—N2—C5—C6	0.7 (2)	C10—C11—C12—C13	1.0 (3)
C7—N1—C6—C5	0.4 (2)	O1—C11—C12—C13	179.3 (2)
C7—N1—C6—C1	178.7 (2)	C8—C13—C12—C11	0.6 (4)
N2—C5—C6—N1	−0.7 (2)	N2—C5—C4—C3	178.5 (2)
C4—C5—C6—N1	177.75 (19)	C6—C5—C4—C3	0.5 (3)
N2—C5—C6—C1	−179.20 (19)	C5—C4—C3—C2	−0.2 (4)
C4—C5—C6—C1	−0.8 (3)	C6—C1—C2—C3	−0.5 (4)
C13—C8—C9—C10	0.7 (3)	C4—C3—C2—C1	0.2 (4)

### Hydrogen-bond geometry (Å, º)

**Table:**

*D*—H···*A*	*D*—H	H···*A*	*D*···*A*	*D*—H···*A*
N2—H2···O2	0.92 (2)	1.86 (3)	2.774 (3)	171 (2)
O2—H2*C*···N1^i^	0.85	2.17	2.876 (2)	140
C15—H15···O1^ii^	0.93	2.56	3.306 (2)	137
C19—H19···N3^iii^	0.93	2.60	3.491 (3)	162

Symmetry codes: (i) −*x*+1/2, −*y*+1, *z*+1/2; (ii) *x*−1/2, *y*, −*z*+1/2; (iii) *x*+1, *y*, *z*.
